# EXPLORING THE CRITICAL CONTRIBUTIONS OF ARTS-BASED APPROACHES IN RURAL DEMENTIA CARE

**DOI:** 10.1093/geroni/igz038.1478

**Published:** 2019-11-08

**Authors:** Rachel Herron

**Affiliations:** Brandon University, Manitoba, Canada

## Abstract

People living with dementia can experience significant barriers to meaningful participation in their communities, particularly in underserviced rural and small-town settings. Drawing on a multi-method pilot study employing observations, diaries, focus groups and interviews in rural Canada, we examine the potential of an innovative dance program developed by Baycrest Health Sciences and Canada’s National Ballet School, to transform the experiences of people living with dementia and the rural places in which they live. Our findings identify moments, processes, and places of transformation throughout the program including moments of individual self-expression; changing interactions with staff, volunteers, and carers; and changing relationships with home and community. We argue that art-based programs can challenge dominant assumptions about people living with dementia and contribute to the creation of more just health and social care in rural places. In doing so, we illustrate the value of critical arts-based approaches to aging in rural places.

